# Protein Phosphatase 2A: Role in T Cells and Diseases

**DOI:** 10.1155/2023/4522053

**Published:** 2023-05-17

**Authors:** Suyasha Roy, Lalit Batra

**Affiliations:** ^1^Immuno-Biology Laboratory, Translational Health Science and Technology Institute, Faridabad, India; ^2^Laboratory of Molecular Immunology, National Heart, Lung, and Blood Institute, National Institutes of Health, Bethesda, MD, USA; ^3^Regional Biocontainment Laboratory, Center for Predictive Medicine, University of Louisville, Louisville, KY, USA

## Abstract

Protein phosphatase 2A (PP2A) is a serine–threonine phosphatase that plays an important role in the regulation of cell proliferation and signal transduction. The catalytic activity of PP2A is integral in the maintenance of physiological functions which gets severely impaired in its absence. PP2A plays an essential role in the activation, differentiation, and functions of T cells. PP2A suppresses Th1 cell differentiation while promoting Th2 cell differentiation. PP2A fosters Th17 cell differentiation which contributes to the pathogenesis of systemic lupus erythematosus (SLE) by enhancing the transactivation of the *Il17* gene. Genetic deletion of PP2A in Tregs disrupts Foxp3 expression due to hyperactivation of mTORC1 signaling which impairs the development and immunosuppressive functions of Tregs. PP2A is important in the induction of Th9 cells and promotes their antitumor functions. PP2A activation has shown to reduce neuroinflammation in a mouse model of experimental autoimmune encephalomyelitis (EAE) and is now used to treat multiple sclerosis (MS) clinically. In this review, we will discuss the structure and functions of PP2A in T cell differentiation and diseases and therapeutic applications of PP2A-mediated immunotherapy.

## 1. Introduction

Protein phosphorylation and dephosphorylation is an important post-translational modification that regulates diverse physiological functions. About 70% of all eukaryotic proteins undergo phosphorylation at serine, threonine, or tyrosine residues by protein kinases [1]. Protein phosphatases antagonize the action of protein kinases acting as a key checkpoint regulator of biological processes. The human genome encodes for around 200 protein phosphatases, which are classified into protein tyrosine phosphatases and protein serine/threonine phosphatases [2]. Protein serine/threonine phosphatases are further subdivided into three subfamilies: phosphoprotein phosphatases (PPPs), aspartate-based phosphatases, and metal-dependent protein phosphatases (PPMs). The PPPs family consists of PP1, PP2A, PP2B, PP4, PP5, PP6, and PP7 [3].

Protein phosphatase 2A (PP2A) contributes to 1% of the total proteins and around 90% of all serine/threonine phosphatase activity in the mammalian cell together with PP1 [4, 5]. In eukaryotes, PP2A is highly conserved across yeasts, drosophila, and mammals with 78%–93% amino acids sequence similarity [6]. PP2A is ubiquitously expressed and integral in the normal functioning and homeostasis of the immune system. PP2A is one of the main factors regulating T cell differentiation and B cell functions [7, 8]. Research over the past two decades has begun to accentuate the involvement of PP2A in T cells, which makes it relevant to emphasize the predominant role of PP2A in T cell biology and T cell-mediated diseases. The pivotal role of PP2A in modulating the optimal functions of B cells has begun to emerge only recently where a study showed that PP2A-deficient B cells displayed diminished germinal center formation and reduced responses to T cell-dependent and T cell-independent antigens [8]. Further research in defining the precise role of PP2A in the immune system besides B and T cells needs to be conducted for better understanding.

This review will provide insights into the basics of structure, physiological functions of PP2A, and its regulation of signal transduction during cellular processes. This review is focused exclusively on the imperative role of PP2A in the activation, differentiation, and functions of T cells. Moreover, the biological role of PP2A in cancer and autoimmune diseases and its implications in immunotherapy has been discussed.

## 2. PP2A: Structure and Functions

PP2A is a highly conserved heterotrimeric serine–threonine phosphatase with a multifarious structure consisting of three distinct subunits—a 65 kD scaffold A subunit (PP2AA), a regulatory B subunit (PP2AB), and a 36 kD catalytic C subunit (PP2AC)—assembled together into a trimolecular complex [5, 9]. The PP2AA and the PP2AC form the “heterodimeric core enzyme” which then associates with the variable subunit PP2AB, forming a complete PP2A holoenzyme [5]. There are two isoforms: *α* and *β*, each of scaffold and catalytic subunits, with *α* isoform being more commonly present than *β* isoform while regulatory B subunit exists in five different isoforms: *α*, *β*, *γ*, *δ*, and *ε* [5].

The catalytic activity of PP2A is regulated by post-translational modifications such as phosphorylation and methylation. The interaction of c-terminus of catalytic subunit of PP2Ac with the PP2A PR55/PR61 regulatory subunit was inhibited due to the phosphorylation of Y307 by receptor-associated tyrosine kinases [10]. Likewise, methylation at L309 by leucine carboxyl methyltransferase 1 (LCMT1) increases the binding intensity of regulatory subunits with the heterodimeric core enzyme (A and C subunits) [11]. PP2A efficacy is also regulated by the autophosphorylation-activated protein kinases which inhibit the auto-protein tyrosine phosphatase activity of PP2A [12].

The PP2A holocomplex takes part in the regulation of key cellular processes, such as cellular metabolism, cell cycle, apoptosis, migration, proliferation, differentiation, and signaling events [13]. Dysfunction in PP2A activity adversely influences the physiological processes and PP2Ac*α* knockout mice are embryonically lethal. Thus, PP2A is indispensable for the normal functioning of cellular pathways. PP2A modulates several signaling cascades, including the phosphatidylinositol-3-kinase (PI3K)/AKT/mammalian target of rapamycin (mTOR) [14, 15], mitogen-activated protein kinase (MAPK) [16], and nuclear factor-kappa B (NF-*κ*B) [17] pathways.

PP2A negatively regulates the AKT pathway by interfering with AKT phosphorylation at threonine-308, which is essential for AKT activation thereby regulating cell survival and proliferation [18]. PP2A has pro-apoptotic activity which is accomplished by inhibiting mitogenic and anti-apoptotic signals via dephosphorylation of MEK1 and ERK-family kinases inhibiting MAPK pathway, negatively regulating PI3K/AKT pathway, suppressing the functions of transcription factors such as signal transducer and activator of transcription 5 (STAT5) and c-MYC, activation of pro-apoptotic factors Bcl-2-associated death protein (BAD) and Bcl-2-interacting mediator of cell death, and inactivation of anti-apoptotic B-cell lymphoma 2 (BCL2) [19–21]. This highlights the role of PP2A as a tumor suppressor which is further supported by the report that PP2A inhibits the protein synthesis of oncogenes such as induced myeloid leukemia cell differentiation protein (MCL1) and c-MYC through dephosphorylation of eukaryotic translation initiation factor 4E (EIF4E) by inhibition of MAPK interacting protein kinases 1 and 2 (MNK1,2 kinases) [22].

PP2A has also shown to negatively regulate wingless/integrated (Wnt) signaling pathway which adversely impacts embryonic development, stem-cell survival, and renewal [23]. This is supported by the fact that inhibition of PP2A augments *β*-catenin-induced transcription, the downstream effector of the Wnt pathway. The B55*α* subunit of PP2A complex directly binds to dephosphorylate and degrades *β*-catenin. This is also attained by the B56*γ*-PP2A complex and glycogen synthase kinase 3 beta (GSK-3*β*) activated by PP2A resulting in the destabilization of *β*-catenin leading to impaired Wnt signaling [24].

There is a myriad of mechanisms through which PP2A regulates the functions of immune cells, particularly T cells. In the next section, the role of PP2A in the activation, differentiation, and functions of T cells has been elaborately discussed.

## 3. PP2A and T Cells

### 3.1. PP2A in T Cell Activation

PP2A has shown to play a key role in T cell activation via multiple mechanisms. PP2A was first described to negatively regulate T cell activation by controlling transmembrane signaling mediated upon TCR engagement [25]. The antigen-specific cytotoxicity potency of lymphocytes was profoundly enhanced upon PP2A inhibition [25]. Another study showed that PP2A dephosphorylates and inactivates AKT by mediating inhibitory CTLA-4 (cluster of differentiation 152) signaling in activated T cells, acting as a potential target for cancer immunotherapy [26]. Zhou et al. [27] demonstrated that shRNA-mediated inhibition of PPP2R2D, a regulatory subunit of PP2A enhances the proliferation, cytokine production, and cytotoxic functions of adoptively transferred effector CD4 or CD8 T cells and tumor-infiltrating lymphocytes (TILs) augmenting antitumor immunity in B16-OVA melanoma model.

Protein phosphorylation and dephosphorylation are essentially required for controlling the switches between different downstream signaling pathways triggered by TCR signaling during T cell activation [28]. On that account, reports suggest that PP2A negatively regulates and suppresses the activation of the NF-*κ*B pathway during TCR signaling [17, 29]. Carma1 acts as a molecular scaffold, bridging the association between TCR signaling to the canonical NF-*κ*B pathway by forming the Carma1-Bcl10-Malt1 (CBM) complex [17]. Carma1 interacts with the PP2A regulatory subunit, PPP2R1A in both resting and activated T cells. PP2A dephosphorylates Ser645 in Carma1, a prerequisite for T cell activation. PP2A inhibition through siRNA knockdown leads to enhanced Carma1 Ser645 phosphorylation augmenting the formation of CBM complex and eventually to NF-*κ*B activation, IL-2, or IFN-*γ* production upon TCR stimulation in T cells [17]. Thus, PP2A limits activation and effector cytokine production in T cells through dephosphorylation of Carma1.

Similarly, it was shown that silencing of B56*γ*, a regulatory PP2A subunit leads to increased phosphorylation of I*κ*B kinase (IKK) and I*κ*B*α* and enhanced NF-*κ*B activity upon TCR stimulation, amplifying the expression of NF-*κ*B target genes such as IL-2 resulting in enhanced T cell proliferation [29]. However, the role of PP2A in synchronizing the development of distinct effector and regulatory T helper cell subsets is invariably different and hence discussed below separately.

### 3.2. PP2A in Th1/Th2 Paradigm

The Th1/Th2 paradigm is mutually exclusive at the molecular level due to the opposing roles of their master transcription factors in their fate. IL-12 and IL-4 program the differentiation of Th1 and Th2 cells through the T-box family of transcription factors (T-bet) and GATA binding protein 3 (GATA3), respectively [30–37]. Although T-bet and GATA3 are the lineage-specific transcription factors required for the development of Th1 and Th2 cells specifically, other signaling components also play a crucial role in deciding their lineage commitment [38]. Among them, mTOR is one of the signaling kinases which regulates the differentiation of Th1 and Th2 cells [39]. Study has shown that mTORC1 is essential for Th1 cell differentiation and mTORC2 was found to be associated with Th2 cell differentiation [39]. Genetic deletion of mTORC1 in CD4^+^ T cells results in reduced phosphorylation of STAT4 in response to IL-12 that leads to impaired differentiation and functions of Th1 cells. Th2 cells can differentiate in the absence of mTORC1 while the presence of mTORC2 activity was required for Th2 cell differentiation [39].

Findings suggest that PP2A activation in lymphocytes results in the suppression of mTORC1 activity while mTORC2 and PI3K/AKT pathways remain unaffected [40]. This provided a strong background that mTORC1 might get activated when PP2A is inhibited in Th1 cells. Consistently, it was shown that pharmacological inhibition of PP2A by LB-100 primed CD4^+^ T cells to differentiate into Th1 cells, remarkably enhancing the production of Th1-related cytokines, such as IFN-*γ*, TNF-*α*, and IL-2 [40]. However, there was an increase in the expression of IFN-*γ*, TNF-*α*, and IL-2 with increasing doses of LB-100 under both Th1 and Th2 skewing conditions [40].

**Figure 1:**
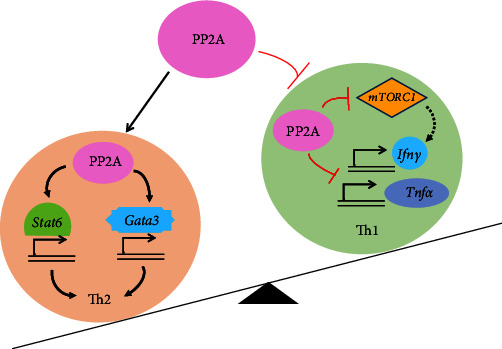
PP2A in Th1/Th2 paradigm. PP2A maintains a reciprocal balance between Th1 and Th2 cells. PP2A binds to the promoter and induces the expression of transcription factors *Stat6* and *Gata3* promoting the differentiation of Th2 cells. In contrast, PP2A inhibits the generation of Th1 cells by restraining the mTORC1 activity and subsequent *Ifnγ* and *Tnfα* gene transactivation.

A previous study has shown that PP2A regulates IL-4-mediated STAT6 signaling [41], which is known to be really important for Th2 cell differentiation. Inhibition of PP2A phosphorylates STAT6 at serine residues which in turn hinders the DNA binding capacity and the transcriptional activity of STAT6 in human CD4^+^ T cells [41]. Furthermore, another study reported that PP2A inhibition significantly abrogated the induction of GATA3 by IL-4 leading to decreased frequency of Th2 cells [40]. Thus, PP2A regulates the paradigm between Th1 and Th2, promoting Th2 while inhibiting Th1 cells (Figure 1).

### 3.3. PP2A in Th17

Transforming growth factor (TGF-*β*1) and IL-6 are required for the differentiation of Th17 cells from naïve CD4^+^ T cells *in vitro* [42, 43]. Th17 cells are defined by the production of effector cytokines such as IL-17, IL-21, and IL-22 and provide protection against extracellular pathogens [44, 45]. However, aberrant Th17 responses may lead to the development of autoimmune diseases due to chronic inflammation [46]. TGF-*β*1 and IL-6 signaling induces ROR*γ*t (RAR-related orphan nuclear receptor (ROR)), which transactivates *Il17a* and *IL17f* gene loci triggering IL-17 expression. ROR*γ*t is the master transcription factor for Th17 cells as mice deficient in ROR*γ*t were found to have impaired Th17 cell development and were partially resistant to experimental autoimmune encephalomyelitis (EAE) [47].

Several reports suggest an association of PP2A with IL-17 expression in T cells. It has been reported that PP2A overexpression is linked with enhanced IL-17 production by CD4^+^ T cells in patients with systemic lupus erythematosus (SLE) [48]. A subsequent study revealed the mechanism behind this observation, where it was shown that the catalytic subunit of PP2A (PP2Ac) promotes the histone 3 acetylation at the *Il17* gene locus facilitating the binding and transactivation of *Il17* gene promoter by the transcription factor interferon regulatory factor 4 (IRF4) [49]. Overexpression of PP2A boosts the inflammatory capacity of T cells by accelerating the chromatin accessibility at the gene loci resulting in increased levels of proinflammatory cytokines as observed in PP2Ac transgenic mice and in human SLE patients [49]. Thus, PP2A promotes the generation of Th17 cells and contributes to the pathogenesis of SLE [49]. PP2A also regulates STAT3 activity which is an absolute requisite for the differentiation of Th17 cells [50]. PP2A dephosphorylates STAT3 at Ser727 residue required for the phosphorylation of STAT3 at Tyr705 in the presence of cytokines leading to the translocation of STAT3 into the nucleus and DNA binding [50]. Consistently, PP2A inhibition increased phosphorylation at Ser727 while decreasing Tyr705 phosphorylation of STAT3 in human Th17 cells [51]. Musculin (MSC), a helix-loop-helix transcription factor upregulates the PPP2R2B, a regulatory member of the PP2A enzyme resulting in the reduced phosphorylation of STAT5B Ser-193 upon IL-2 triggering in human Th17 cells [51]. Thus, Musculin leads to higher levels of PPP2R2B and impaired induction of IL-2-dependent target genes, inhibiting Th17 response to IL-2 signaling by restraining STAT5B activity [51].

**Figure 2:**
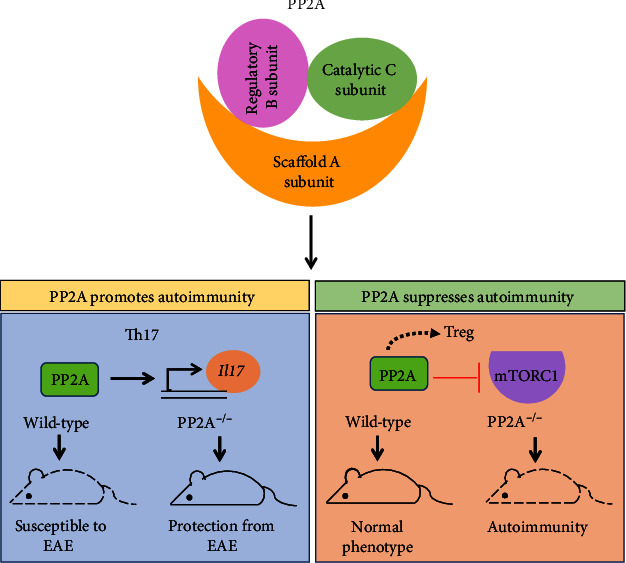
PP2A in T cell-mediated autoimmunity. PP2A is a serine/threonine phosphatase composed of trimolecular complex of scaffold subunit A, regulatory subunit B, and catalytic subunit C. PP2A promotes Th17 cell differentiation and increases *Il17* expression contributing to the development of EAE as PP2A^−/−^ mice were protected from EAE. Contrarily, PP2A^−/−^ mice showed severe autoimmunity due to impaired immunosuppression by Tregs.

Further, it was shown that the deficiency of PP2A abolished the C-terminal phosphorylation of SMAD2 (mothers against decapentaplegic 2) but increased the C-terminal phosphorylation of SMAD3 (mothers against decapentaplegic 3). This reduces the expression of *Il17* gene transcription by altering ROR*γ*t activity resulting in decreased Th17 cell differentiation and renders protection to PP2A^−/−^ mice from EAE [52]. Thus, PP2A could be a potential candidate for targeting Th17-mediated autoimmune diseases (Figure 2).

### 3.4. PP2A in Tregs

Regulatory T cells (Tregs) express the transcription factor Foxp3 and is involved in the elimination of autoreactive T cells in the periphery maintaining immune homeostasis [53]. Tregs display unique cytokine, metabolic, and proliferation profiles [54]. Owing to the immunological tolerance induced by Tregs, the deletion of immunosuppressive functions of Tregs results in severe autoimmunity in both mice [55] and humans [56]. The activation and maintenance of Tregs require the constant involvement of TCR, CD28, and IL-2 signaling, as a defect in any of these signals leads to severe impairment of suppressive functions of Tregs [57–59]. Paradoxically, Tregs exhibits downregulated activity of signaling pathways such as metabolic-checkpoint mTOR [38, 60] and the PI3K/AKT pathways [61].

PP2A has also shown to play an important role in fine-tuning the development and functions of Tregs. PP2A activity was found to be elevated in Tregs as compared to conventional T cells in which PP2A is inactivated by Tyr307-phosphorylation upon TCR stimulation [62]. Apostolidis et al. [62] reported that PP2A inhibits mTORC1 complex in Tregs which is essential for their immune suppressive functions. Mechanistically, transcription factor Foxp3 represses Sgms1 (encoding sphingomyelin), resulting in ceramide accumulation in Tregs. Higher levels of ceramide inhibit SET (endogenous inhibitor of PP2A) allowing the enhanced PP2A activity in Tregs [62]. Hence, ceramide triggered increased PP2A activation restrains the mTORC1 complex in Tregs. Subsequently, genetic deletion of PP2A in Tregs resulted in impaired immunosuppressive capability due to increased mTORC1 signaling which contributes to a severe, multiorgan, lymphoproliferative autoimmune disorder [62].

It has also been shown that the differentiation of Tregs is significantly impaired by mTORC1 hyperactivation in naïve CD4^+^ T cells [40]. Pharmacological inhibition of PP2A by LB-100 hyperactivates mTORC1 signaling which could inhibit Treg development. Consistently, it was shown that increasing doses of LB-100 suppressed the induction of forkhead box P3 (Foxp3), master transcription factor of Tregs, by TGF-*β* [40]. A recent study further substantiates the above findings where it has been reported that TGF-*β*1 inhibits mTORC1 signaling resulting in enhanced activation of PP2A [63]. mTORC1 signaling was suppressed when natural Tregs was treated with TGF-*β*1 leading to downregulation of glycolytic activity while PP2A gets activated [63]. PP2A activation is required for the maintenance of Foxp3 expression induced by TGF-*β*1 since the inhibition of PP2A with okadaic acid treatment disrupted the Foxp3 expression in natural Tregs [63].

PP2A promotes Tregs by potentiating IL-2R signaling through multiple mechanisms. First, PP2A cooperates with CD25 enhancing IL-2R signaling leading to the tyrosine phosphorylation of Janus kinase 3 (JAK3) and STAT5 in human Tregs [64]. Consistently, genetic ablation of PP2A in human and mouse Tregs disrupted IL-2-mediated STAT5 activation, an absolute requisite for Treg development and functions [64]. Overexpression of PP2A profoundly upregulated the expression of proteins involved in the activation, functions, and survival of Tregs [64]. Next, PP2A maintains IL-2R signaling by preventing the loss of surface IL-2*β*R in thymus- and spleen-derived Tregs by inhibiting the sheddase activity of selective A disintegrin and metalloproteinase 10 (ADAM10) [65]. Forced expression of IL-2*β*R or ADAM10 inhibition in PP2A-deficient Tregs restores the competent IL-2R signaling required for maintaining the Foxp3 expression and progression of Tregs [65]. Cumulatively, these studies reinforce the critical role of PP2A in the induction and immunosuppressive functions of Tregs (Figure 2).

### 3.5. PP2A in Th9

Th9 cells arise from the differentiation of naïve CD4^+^ T cells in the presence of TGF-*β*1 and IL-4 [66, 67] and play an important role in allergic inflammation [68] and antitumor immunity [69, 70]. Transcriptomics studies have revealed an important role of several transcription factors in the development of Th9 cells and IL-9 induction [66, 67, 71, 72]; however, the role of post-translational modifications in the expansion of Th9 cells remains unknown. Recently, it was shown that there was an abundance of phosphoproteins in the proteome of Th9 cells, among which PP2A was highly upregulated [73]. PP2A inhibition led to impaired differentiation of Th9 cells by downregulating the key transcription factors of Th9 cells such as *Spi1*, *Irf4*, *Gata3*, *Batf*, *Irf1*, *Hif1α*, and *Foxo1* [73]. Consistently, the siRNA-mediated knockdown of PP2A significantly inhibited IL-9 induction which functionally validated the positive regulation of Th9 cell differentiation by PP2A [73]. Further, the inhibition of PP2A significantly abrogated the antitumor immune response of Th9 cells by decreasing the frequency of IFN-*γ*-producing cytotoxic CD8^+^ T cells in the spleen, tumor-draining lymph nodes and tumor-infiltrating lymphocytes in B16-OVA melanoma model [73]. Thus, PP2A plays a critical role in the differentiation and antitumor functions of Th9 cells. However, the precise mechanism through which PP2A controls the expression of key transcription factors and consequently IL-9 induction in Th9 cells needs further elucidation.

## 4. PP2A and Diseases

PP2A has shown to be involved in the development of cancer [74], SLE [75], and neurodegenerative diseases such as multiple sclerosis [76]. In addition to the involvement of PP2A in T cell-mediated diseases, PP2A has shown to play an important role in diseases independent of T cell activity such as Parkinson's disease, Alzheimer's disease, and type I autoimmune diabetes [77–83]. In view of the physiological relevance of PP2A and its pivotal role in T cell differentiation, it is extremely important to shed light on the contribution of PP2A and their regulation of T cells in the context of inflammation and diseases, as discussed below.

### 4.1. PP2A in Cancer

Global phosphorylation mainly involves serine, threonine, or tyrosine phosphorylation acting as on–off switch in cellular signaling. Aberrant phosphorylation characterized by unusual activation of protein kinases leads to anomalous activation of signaling pathways resulting in malignant transformation [24]. Protein phosphatases keep a check on these molecular networks by negatively regulating such cancer-promoting signals. PP2A, the most abundant serine/threonine protein phosphatase in humans, targets oncogenic kinase and its downstream effectors and kills cancer cells while sparing normal cells. Restoration of PP2A activity in leukemic T cells results in significant impairment in the proliferation and survival of cancer cells in an oncogene-independent manner [24].

Dysfunction of PP2A activity due to aberrant expression and mutations in the regulatory and scaffold subunits has shown to be associated with various human cancers, highlighting its role as a “tumor suppressor” [3]. It was first reported two decades ago that PP2A inhibition by okadaic acid promotes tumor growth [84]. In parallel, another study showed that the regulatory subunits of PP2A were displaced by Simian Virus 40 (SV40) small T (ST) antigen and murine polyomavirus middle T (MT) antigen rendering the phosphatase activity of PP2A nonfunctional contributing to neoplastic transformation [85]. These two preliminary evidences strengthened the tumor suppressive role of PP2A. Further, endogenous cellular inhibitors such as cancerous inhibitor of PP2A (CIP2A) and inhibitor 2 of PP2A (I2PP2A; commonly known as SET) restrains PP2A activity and are often found to be overexpressed in cancer [24, 86–89]. Consistently, PP2A-activating drugs, such as FTY720 (fingolimod) and its derivatives, effectively restore tumor-suppressor activity of PP2A and antagonize the tumor progression [24]. Thus, pharmacological and cellular inhibitors of PP2A and PP2A-activating drugs are emerging as key players in cancer surveillance.

Immune-based cancer therapies have garnered strong attention in recent years by virtue of their diverse strategies to activate the patient's immune system to fight against cancer cells. This “host versus tumor” effect involves the use of monoclonal antibodies (mAbs) targeted against checkpoint regulators such as CTLA-4, PD-1 (programmed cell death protein 1) on T cells, vaccines directed against cancer antigens, targeting immunosuppressive molecules, and autologous chimeric antigen receptor (CAR) T cell therapy [90–92]. The PI3K/AKT axis remains the central node for regulating the functions of T cells by PP2A [93, 94]. Checkpoint blockade therapy targeting CTLA-4 and PD-1 on T cells has shown efficacy in the treatment of leukemia and solid tumors such as melanoma [90, 91]. Upon ligation with PD-L1/PD-L2 and CD28, PD-1 and CTLA-4 represses AKT signaling, respectively; CTLA-4 works by directly activating PP2A in T cells through an unknown mechanism [26]. Another report demonstrated that CIP2A deficient mice harbored lower frequency of both CD4^+^ and CD8^+^ effector T cell populations suggesting that PP2A inhibition is required for the adaptive immune response [95]. Moreover, it has been shown that combinatorial immunotherapy using LB-100, a PP2A inhibitor together with PD-1 blockade further enhances CD4^+^ and CD8^+^ T cell-mediated antitumor immune responses in mouse melanoma model without inducing autoimmunity [40]. This reflects a translational potential of LB-100 in combination with PD-1 blockade in the treatment of solid tumors. Thus, exploiting agents that target PP2A in cancer as combination therapy has a potential role in inducing antitumor immunity [96–98].

### 4.2. PP2A in Systemic Lupus Erythematosus

Systemic lupus erythematosus (SLE) is an autoimmune disease characterized by the altered antigen receptor-mediated T cell activation. PP2A dysregulation may contribute to SLE and increased PP2A levels has been linked to autoimmunity in SLE patients and transgenic mice [49]. Importantly, SLE has shown to be associated with higher levels of PP2A which tightly regulates the production of IL-2 and IL-17 by CD4^+^ T cells and controls T cell apoptosis triggered by IL-2 deficiency [75, 99].

Patients with SLE have higher levels of catalytic subunits and activity of PP2A resulting in decreased IL-2 production in T cells [99]. The reduction in IL-2 levels in SLE T cells occurs through different mechanisms. First, the hypomethylation within the cAMP response element (CRE) region enhances the binding of p-CREB to the CRE site leading to increased PP2A activity resulting in diminished IL-2 production in SLE T cells [100]. Second, in SLE patients, higher levels of PP2A drive the dephosphorylation of the transcription factor Elf-1, inhibiting the binding of Elf-1 to the CD3*ζ* and FcR*γ* promoters. As a result, the levels of CD3*ζ* chain go down while the FcR*γ* chain goes up within the CD3 complex leading to aberrant CD3-TCR signaling and reduced IL-2 production in SLE T cells [101]. Notably, IL-2 production increases with the replenishment of the CD3*ζ* chain expression in SLE T cells [102]. Third, aggravated PP2A activity dephosphorylates the transcription factor specificity protein-1 (SP-1) at Ser59 which then strongly binds to the basic leucine zipper transcription factor cAMP-responsive element modulator *α* (CREM*α*) [103]. Consequently, the unusual higher levels of CREM*α* bind and subdue the transactivation of IL-2 and T cell receptor *ζ* chain promoters resulting in diminished IL-2 production in SLE T cells [103].

Besides suppressing IL-2 production, elevated levels of PP2A dephosphorylates MEK and ERK in response to T cell activation, abrogating the enzymatic activity of DNA methyltransferase (DNMT) which hinders the DNA methylation essential for optimal T cell activation [104]. Thus, DNA hypomethylation serves as a characteristic feature in SLE patients. Further, gene expression analyses have shown that IL-17 production was accelerated with increased PP2A expression in T cells. In both PP2A transgenic mice and SLE patients, it has been demonstrated that PP2A increases the histone 3 acetylation at the *Il17* gene locus and activates the transcription factor IRF4 in T cells resulting in an exaggerated immune response [49]. Therefore, inhibition of PP2A could be a potential therapeutic strategy for preventing the development of Th17-mediated autoimmune disorders such as SLE. In contrast, PP2A-deficient Tregs in mice lead to the manifestation of severe lymphoproliferative and autoimmune disorders due to the autoantibody production against lupus-associated nuclear autoantigens. Mice with deficiency of PP2A in Tregs showed enhanced activation of CD4^+^ and CD8^+^ T cells and consequently increased production of IL-17, IL-2, IFN*γ*, and TNF*α* [62, 105]. Thus, PP2A-driven immunosuppressive functions of Tregs could be exploited for controlling autoimmunity *in-vivo*.

### 4.3. PP2A in Multiple Sclerosis

Multiple sclerosis (MS) is a chronic inflammatory autoimmune disease that is characterized by the destruction of myelin sheaths of neurons by autoreactive lymphocytes causing neurological dysfunction [106]. Reports have shown that FTY720, a PP2A-activating drug reduced neuroinflammation in experimental autoimmune encephalomyelitis (EAE), a mouse model of multiple sclerosis [107], and is clinically used to treat the relapsing form of MS [108]. The mechanism behind the remission of MS has been attributed to the anti-inflammatory effects of FTY720 by blocking the egress of autoreactive T cells from lymph nodes and hindering the infiltration of these self-reactive T cells into the CNS causing peripheral lymphopenia [109]. In addition, FTY720 inhibits the secretion of proinflammatory cytokines and T cell proliferation by impairing the functions of dendritic cells (DCs), thereby diminishing the DC-mediated T cell functions [110, 111].

In line with these studies, it has been shown that FTY720 impairs Th1 effector response by inhibiting the IL-12 production by activated DCs [76]. Moreover, FTY720 also abrogates DC-mediated Th2 effector responses as demonstrated in an experimental mouse model of asthma [112]. Thus, exploiting strategies for PP2A activation would be an excellent measure in controlling T cell-mediated neurodegeneration in multiple sclerosis.

## 5. PP2A in Immunotherapy

Considering the importance of PP2A in the regulation of physiological functions and its dysregulation in diseases, there is a budding interest in the development of PP2A-directed therapeutics. The main underlying strategies that are being exploited in the PP2A-directed therapeutics include direct targeting of PP2A, suppressing the endogenous PP2A inhibitors, or hindering the post-translational modifications on PP2A subunits [113]. Multiple clinical trials have been conducted and are ongoing based on PP2A-directed therapeutics in the treatment of cancer and neurological disorders. LB-100 (PP2A inhibitor) has shown promising efficacy with the completion of phase I clinical trial in the treatment of patients with relapsed solid tumors through therapeutic modulation [114, 115]. Currently, the safety and efficacy of LB-100 is being tested for the treatment of glioblastoma (a phase II clinical trial), myelodysplastic syndrome (a phase I/II clinical trial), and small-cell lung cancer (a phase I clinical trial) [115]. LB-100 alone, or in combination with standard chemotherapy drugs such as carboplatin, etoposide, and atezolizumab, is being administered in patients with extensive-stage small-cell lung cancer in phase I clinical trial, with the speculation that this combinatorial therapy could improve the existing treatment regimen [115]. This is based on the rationale that by blocking PP2A, LB-100 promotes the tumor cells to divide which allows the better killing of dividing tumor cells by the chemotherapy drugs. As a therapeutic strategy, inhibition of PP2A enhances the sensitization of cells to immunotherapy and treatment of autoimmune diseases and cancer, while tumor suppressive functions of PP2A is boosted by exploiting PP2A-activating strategies [116]. The multimeric nature of PP2A creates an obstacle in successfully designing modulators although it does help in developing selective agents for chemically targeting PP2A. Therefore, detailed characterization of the heterotrimers and their biological functions will help to design effective strategies targeting PP2A in immunotherapy.

## 6. Future Perspectives

PP2A illustrates the prototype of a highly regulated protein phosphatase family with inherent tumor suppressive properties. PP2A is responsible for constraining inflammation by maintaining a proper balance of the signaling events and offers immense potential by mounting immunomodulatory effects during pathophysiology. It is well appreciated that PP2A suppression unleashes oncogenic signals triggered by unrestrained activity of kinases. This attribute fosters PP2A as an attractive therapeutic target to improve clinical outcomes in devastating malignancies. Though understudied, the knowledge of the role of PP2A in T cell biology is growing with the years. PP2A has shown to tightly control the signaling pathways in T cells which is accountable for sustained immune response during homeostasis and inflammation. A more comprehensive understanding of the role of PP2A in T cell subsets will empower the strategy for targeting PP2A with inhibitors or stimulators under the given circumstances. This will contribute to the development of enhancers or inhibitors of PP2A in the treatment of T cell-mediated tissue inflammation such as SLE and other autoimmune diseases. We anticipate that in the coming years, PP2A-directed therapeutic strategies will be more appreciated with the increased attainment of FDA approval for PP2A-targeting drugs. PP2A-driven successful and ongoing clinical trials for the treatment of cancer have paved the way for the development of immunotherapeutic strategies for alleviating autoimmune disorders. Preclinical validation of drugs in disease models for autoimmunity coupled with PP2A-targeting drugs for immunooncology will potentially also lead to the inception of clinical trials for PP2A-targeting agents in the treatment of autoimmune diseases. Thus, PP2A is an intriguing target that could be exploited in synchronizing T cell-mediated autoimmunity and antitumor immune responses, mitigating cancer and autoimmune diseases.
